# Non-Destructive Detection of Fruit Quality: Technologies, Applications and Prospects

**DOI:** 10.3390/foods14122137

**Published:** 2025-06-19

**Authors:** Jingyi Liu, Jun Sun, Yasong Wang, Xin Liu, Yingjie Zhang, Haijun Fu

**Affiliations:** 1 School of Electrical and Information Engineering, Jiangsu University, Zhenjiang 212013, China; 2 School of Automotive and Traffic Engineering, Jiangsu University, Zhenjiang 212013, China

**Keywords:** fruit quality, non-destructive testing, near-infrared spectroscopy, hyperspectral imaging, electronic nose

## Abstract

Fruit quality testing plays a crucial role in the advancement of fruit industry, which is related to market competitiveness, consumer satisfaction and production process optimization. In recent years, nondestructive testing technology has become a research hotspot due to its outstanding advantages. In this paper, the principle, application, advantages and disadvantages of optical, acoustic, electromagnetics, dielectric properties research and electronic nose non-destructive testing technology in fruit quality testing are systematically reviewed. These technologies can detect a variety of chemical components of fruit, realize the assessment of maturity, damage degree, disease degree, and are suitable for orchard picking, quality grading, shelf life prediction and other fields. However, there are limitations to these techniques. The optical, acoustic and electronic nose technologies are susceptible to environmental factors, the electromagnetic technology has defects in the detection of complex molecules and fruit internal quality, and the dielectric characteristics are greatly affected by the shape and state of the sample surface. In the future, efforts should be made to enhance the implementation of non-destructive testing technology in the fruit industry through technology integration, optimization algorithm, cost reduction, and expansion of industrial chain application, so as to help the premium growth of the fruit industry.

## 1. Introduction

The quality of fruit, an integral part of people’s daily diet, is closely linked to consumers’ health and satisfaction [1,2]. During the advancement of the fruit industry, there are stringent demands for precisely detecting and managing fruit quality at every stage, from orchard cultivation to market distribution [3,4]. With the continuous growth of the global fruit trade and the rising consumer expectations for taste, freshness and safety of fruits, traditional fruit quality assessment techniques, such as sensory evaluation [5,6], physical and chemical analysis [7,8], have been unable to fulfill the demands of rapid, accurate and non-destructive evaluation in the modern fruit industry due to the limitations of strong subjectivity, destructive detection process and low efficiency.

Non-destructive testing technology has become a focal point and development trend in the domain of fruit quality testing, owing to its significant advantages of not destroying the original structure and characteristics of fruit samples and not affecting the subsequent sales and use value of fruits [9,10,11]. In recent years, a variety of non-destructive testing techniques, such as optical technology, acoustic technology, electromagnetic technology, dielectric properties technology and electronic nose technology [12,13,14,15], have made great progress in fruit quality detection and have been widely used. By interacting with substances in fruits, these technologies can obtain various information reflecting fruit quality, such as content of components, hardness, maturity, damage and disease conditions, which provides powerful technical support for fruit planting management [16], postharvest treatment [17], storage and transportation [18], and marketing. For consumers, non-destructive testing of fruit quality not only ensures food safety [19], but also links the information of fruit quality with consumers’ sensory parameters and preferences [20,21], thereby enhancing consumers’ satisfaction with fruits.

This paper aims to systematically review the principles, application results, advantages and limitations of these non-destructive testing technologies in fruit quality testing. Through comprehensive analysis and comparison, it provides comprehensive and in-depth technical reference for relevant practitioners in the fruit industry, promotes the further development and utilization of non-destructive fruit testing technology, and promotes the high-quality advancement of the fruit industry.

## 2. Optical Technology

### 2.1. Near-Infrared Spectroscopy

Near-infrared spectroscopy (NIRS) emerged in the 1970s and was first applied to detect the water and protein content in grains. It was greatly developed in the mid-to-late 1980s [22], and has now become a very widely used nondestructive testing method. The principle is that when near-infrared light interacts with the sample, different chemical components in the sample will absorb particular wavelengths of near-infrared light selectively through the quantized energy level transition mechanism due to their unique molecular vibration frequencies. After the characteristic response of the interaction between light and matter is captured by the detector, the spectral information reflecting the composition characteristics of the sample can be obtained through analysis [23,24]. The wavelength range of NIRS is approximately 780–2500 nm [22].

**Figure 1:**
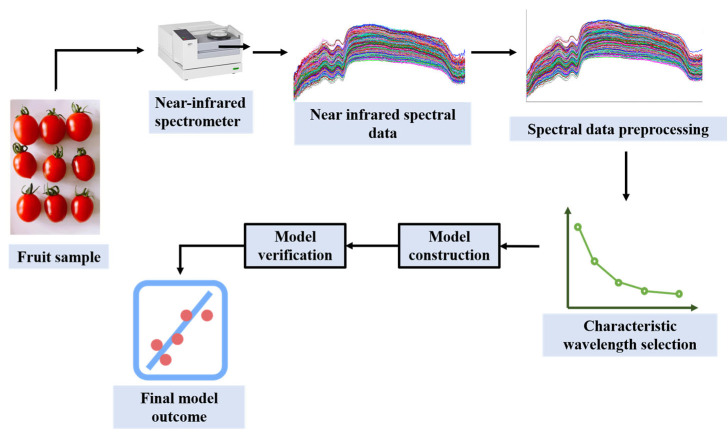
The process of using NIRS technology to detect fruit quality (redesigned from Ref. [27]).

When analyzing a component of a sample using NIRS techniques, NIRS data, reference data for that component, and calibration model are usually required. By establishing a suitable calibration model, the spectral data can be close to the reference data, thus achieving the purpose of accurately predicting the specific components in the sample [25]. In application, NIRS data usually need to go through pre-processing, feature extraction and other steps, and then a model should be built [26]. The process of fruit quality detection by NIRS is shown in Figure 1.

At present, NIRS is often used to detect indicators such as sugar content, soluble solid content (SSC) and acidity in fruits, so as to complete the prediction of fruit maturity, disease degree and consumer preference under non-destructive conditions, providing a strong basis for reflecting of fruits quality.

In the aspect of fruit quality inspection, SSC remains a key research focus. Many literatures have demonstrated the feasibility of NIRS in the detection of fruit SSC. For example, Urraca et al. [28] found that using a portable NIRS analyzer to detect SSC of grape berries, the predictive error (RMSEP) of the calibration model could reach 1.42°Brix (Brix, representing grams of sucrose per 100 g solution at 20 °C) under laboratory conditions. Under field conditions, it was 1.68°Brix. A prediction model was constructed by Bai et al. [29] for the SSC of apples from various producing areas by means of spectral fingerprint feature extraction, deep learning and other technologies, and its Rp reached 0.990 and RMSEP was 0.274, which significantly improved the prediction accuracy. In addition, the technology also enable the detection of other fruit quality indicators. For example, Ferrer-Gallego et al. [20] applied NIRS to assess sensory parameters (taste, texture, visual and olfactory properties) of grape seeds and skins and found that this technique has great potential for predicting these properties; Daniels et al. [30] applied fourier transform NIRS (FT-NIRS) to detect internal ripening parameters of complete grape clusters, including SSC, titratable acidity (TA), sugar-acid ratio, providing a new method for grape quality assessment.

In terms of technical optimization, many studies have focused on spectral preprocessing techniques and variable selection methods. Yuan et al. [31] contrasted different spectral preprocessing methods and found that Savitzky-Golay filtering combined with multiple scattering correction (MSC) had a better effect in apple spectral processing; Sun et al. [27] adopted the iterative retained information variable (IRIV) algorithm to decrease the dimension of spectral data of cherry tomato, productively obtaining the characteristic wavelengths and optimizing the model’s prediction accuracy. Optimization of the detection probe and experimental parameters can also lead to an enhancement of the detection effect. Yu et al. [32] designed a detection probe and optimized the relevant parameters, enabling the grape SSC prediction model based on near-infrared diffuse transmission spectroscopy to achieve better results.

Model construction and improvement are also important research directions in the application of NIRS for fruit quality detection. Partial least squares (PLS) is a frequently utilized modeling approach. However, to improve the model performance, scholars have tried various improvement strategies. Els et al. [33] utilized NIRS combined with PLS to estimate SSC and hardness of apples from diverse varieties, from various origins and with different storage times; Guo et al. [34] compared linear versus nonlinear regression models and found that the independent component analysis-support vector machine (ICA-SVM) model performed better in predicting apple SSC; Bai et al. [29] combined deep learning with spectral fingerprint features to construct a multi-origin apple SSC prediction model, markedly enhancing the model’s generalizability and prediction accuracy.

The NIRS detection method is characterized by its high speed performance [35,36,37,38] and extensive applicability [20,21,27,28,29,30,31,32,33,34,35,36,37,38,39,40,41,42]. Some studies use portable (handheld) NIRS analyzers [28,31], which can quickly complete the spectral collection of fruits in fields and other scenarios and have high controllability. Meanwhile, NIRS can contain rich sample information, fully saving detection time. Nevertheless, the detection outcomes of NIRS are vulnerable to biological variability. For instance, the spectra of different apple varieties vary significantly near the absorption peaks of water (at 970, 1170, and 1450 nm), which affect the prediction accuracy of their SSC and hardness [33]. Furthermore, traditional NIRS devices are temperature-sensitive. Changes in sample temperature can affect the absorption and peak positions of the spectrum, thereby influencing the measurement accuracy [36]. In actual detection, NIRS has high requirements for the uniformity of samples (such as size and shape) and surface conditions (such as no dust and no defects) [21,37,40]. Therefore, operators need to select and pretreat fruit samples to reduce the impact on predictive performance. In addition, the rich information contained in NIRS may cause severe spectral overlap, causing challenges in determining the content of each component from the spectrum [21]. The prediction models established by NIRS technology often have poor universality and require the establishment of corresponding models for different fruits under different conditions, which has high requirements for operators in terms of data processing and analysis.

### 2.2. Hyperspectral Imaging

After emerging in the 1980s for remote sensing, hyperspectral imaging (HSI) was later adopted for agricultural and food research [43,44]. It is usually capable of capturing near-infrared, visible light and short-wave infrared spectra within the range of 400–2500 nm [44]. By combining spectroscopy with imaging techniques, HSI is able to retrieve the spectral characteristics of objects in multiple consecutive narrow bands, generating a three-dimensional data cube that incorporates two-dimensional spatial information and one-dimensional spectral information [45]. Essentially, HSI technology capitalizes on the variations in how substances absorb and scatter light at various wavelengths. When light interacts with matter, the molecules in the matter absorb light of specific wavelengths. This is because the energy level transitions such as vibration and rotation of the molecules match the energy of specific wavelengths of light. The molecular structures of different substances vary, and so do their characteristics of absorbing and scattering light, thus generating unique spectral features. Therefore, they can serve as the basis for identifying and analyzing substances.

**Figure 2:**
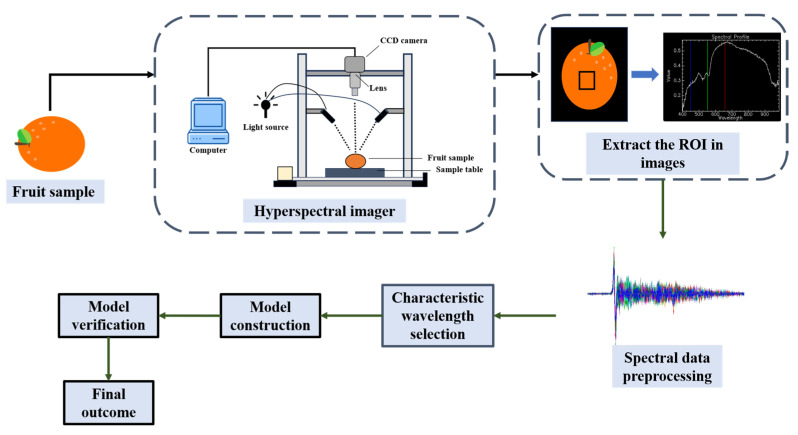
The process of detecting fruit quality using HSI technology (redesigned and redrawn from Ref. [52]).

The three-dimensional data obtained by HSI usually need to undergo reflectance calibration, that is to say, it is essential to consider the white background response and black background response of the instrument (the camera response recorded when the light source is switched off and the lens is fully enclosed by a light-tight cover) [46]. In the process of handling these three-dimensional data, methods such as image calibration, image enhancement and segmentation are also required [47] to extract the region of interest (ROI) in the image and obtain the average spectral curve of ROI. Similar to near-infrared spectroscopy, the collected spectral curves usually require preprocessing (such as baseline correction and normalization) and dimensionality reduction processing (such as principal component analysis (PCA) and successive projection algorithm (SPA)) to improve data quality and analysis efficiency [43,48,49,50,51], and then researchers can construct models to predict the components in the samples. The process of fruit quality detection by HSI technology is shown in Figure 2.

HSI technology is suitable for non-destructive evaluation studies in various fruit varieties. Chen et al. [53] applied near-infrared hyperspectral imaging (NIR-HSI) technology with chemometrics approaches to conduct quantitative predictions of sugar (SU), vitamin C (VC), and organic acid (OA) in pomelo. Xiang et al. [54] developed a non-destructive testing technique for detecting the SSC and hardness of cherry tomatoes drawing on the HSI technology of 400–1000 nm and a novel one-dimensional convolutional residual network (Con1dResNet) regression model. Xu et al. [55] utilized HSI techniques ranging from 400 to 1001 nm, and combined the stacked autoencoder (SAE), PLS, and least squares support vector machine (LS-SVM) in deep learning algorithms, to estimate the SSC and TA of Kyoho grapes.

In the detection of fruit freshness, HSI technology shows many advantages. Li et al. [52] utilized HSI technology and achieved effective detection of early rotten oranges by constructing models such as Linear Partial Least Squares Discriminant Analysis (PLS-DA) and nonlinear backpropagation artificial neural network (BP-ANN). Mousavi et al. [56] employed HSI along with multiple deep learning networks such as ResNeXt and RegNetX to classify healthy pomegranates and frozen pomegranates. The outcomes indicated that the precision of every model exceeded 99%, among which the EfficientNetV2 model performed the best. Shi et al. [57] utilized HSI technology, integrating multi-objective feature selection and multi-task models, to simultaneously detect the preservation conditions and storage period of yellow peaches. The results indicated that HSI method not only reduced the computational cost by 66.67% but also improved the accuracy rate.

Regarding the detection of minor damage to fruits, Zhao et al. [58] utilized HSI data within the range of 500–900 nm, extracted characteristic images at a wavelength of 547 nm through PCA, and then applied non-uniform quadratic difference and image processing techniques. The detection accuracy rate for minor damage to apples reached 88.57%. Baranowski et al. [59] used equipment including visible and near-infrared (400–1000 nm), short-wave infrared (1000–2500 nm) hyperspectral cameras and mid-wave infrared (3500–5000 nm) thermal imaging cameras to conduct systematic research on the early damage of apples. Through PCA and minimum noise fraction (MNF) analysis, researchers can identify the defect areas and healthy areas of fruit tissues. After pulsed heating of the fruit surface, Fast Fourier transform (FFT) is applied to the image sequence to obtain the location and depth information of damaged tissue.

In terms of detecting external pests of fruits, Liu et al. [60] established a 900–1700 nm NIR-HSI system to inspect external pests of jujubes. Based on PCA and the ratio of two bands, they combined the image subtraction algorithm to develop a detection algorithm. The recognition accuracy rate of pest-infested jujubes reached 93.1%, and the classification rate of intact jujubes was 100%. The feasibility of HSI technology in detecting external pests of jujubes has been proved.

HSI technology, with its unique detection principle and data acquisition and processing method, has the ability to obtain massive information and can present the external and internal characteristics of fruits in all aspects [52,53,55,56,57,58,59,60,61,62,63,64,65,66,67,68]. Meanwhile, HSI technology can achieve high-precision detection [52,56,57,61,65] and also has certain advantages in detection speed. However, the HSI technology, similar to NIRS, obtains an abundant amount of spatial and spectral information, which also increases the difficulty of data processing. Often, methods such as preprocessing and feature selection [52,53,55,56,57,58,59,60,61,62,63,64,65,66,67,68] are required to enable the established model to have a higher accuracy rate. Meanwhile, hyperspectral imaging technology is also vulnerable to environmental factors. For example, when applied in orchard environments, factors such as light, temperature and terrain will all pose challenges to HSI detection [64]. In addition, HSI systems usually contain high-performance CCD cameras, imaging spectrometers and other equipments [54,60] which have a relatively high cost. To some extent, the relatively high cost of HSI hinders the popularization and utilization in actual production.

### 2.3. Visible Light Imaging

**Figure 3:**
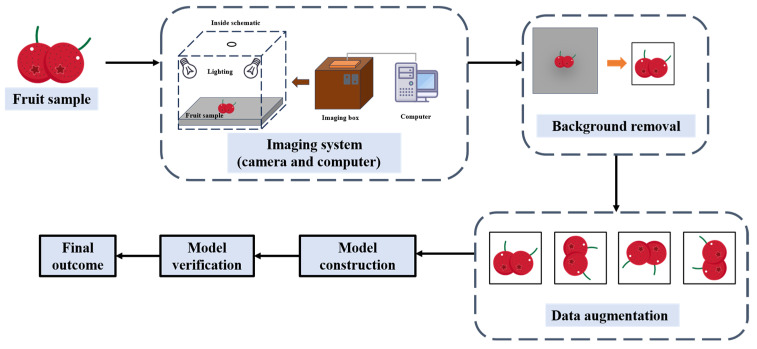
The process of detecting the quality of fruits using visible light imaging technology (redesigned and redrawn from Ref. [77]).

Visible light imaging is a detection technology simulating human vision [69]. By obtaining images through cameras and sensors, it can present details like the shape, color and surface defects of samples, thereby achieving the purpose of predicting quality [70]. Visible light imaging technology can be installed on different devices and applied to various stages of fruit growth [71], such as fruit-picking robots in orchards [72,73,74], post-harvest fruit grading and sorting [70], disease detection [75]. Visible light imaging is closely related to the field of artificial intelligence. For example, visible light imaging technology based on convolutional neural network (CNN) is widely used in the external quality examination of fruits [76], and has also become a continuous driving force for the development of the field of visible light imaging for fruit quality inspection. The process of fruit quality detection by visible light imaging technology is shown in Figure 3.

Visible light imaging has achieved remarkable results in the domain of fruit recognition and classification. Liu et al. [78] proposed an approach relying on block classification for the identification of apples within plastic bags. Firstly, the watershed algorithm based on R-G grayscale image edge detection was employed to segment the image, reducing the number of blocks and retaining the fruit edges. Then, SVM was used for classification utilizing color and texture characteristics, effectively suppressing light interference. The Internet of Things ultrasonic and visual automatic fruit sorting system developed by Huynh et al. [79] integrates multiple components and can sort various fruits into four customizable trays based on fruit size and color classification, with an accuracy rate of 90% and good scalability. Zhang et al. [80] constructed a multi-class detection model for cherry tomatoes based on the improved YOLOv4-Tiny, classified cherry tomatoes into four categories, and improved the detection accuracy by introducing FENB and FEN with the CSPNet structure. For the test images captured during daytime and nighttime, the detection precision of cherry tomatoes under various occlusion conditions was considerable. The mAPs were 90.78% and 94.72% respectively, which were superior to the YOLOv4 and YOLOv4-Tiny models.

In the field of fruit appearance inspection, Nazrul et al. [81] utilized computer vision and deep learning technologies to build a low-cost real-time vision inspection system based on Raspberry PI and trained multiple models. For the apple and banana test sets, the EfficientNet model obtained average accuracy rates of 99.2% and 98.6% respectively, and it also had a relatively high accuracy rate in real-time tests, achieving the automatic detection of fruit appearance. Jaime et al. [82] employed models such as Mask R-CNN to segment pomegranate fruits and proposed a fruit size estimation algorithm. This algorithm has high accuracy, with a median relative error of 1.39%. Qiu et al. [83] constructed a visible light imaging system to collect apple images and classified the images using classic CNN models. The VGG16 model performs the best, providing a new method for apple grading. Gu et al. [84] introduced a new algorithm to quantify texture features in the detection of watermelon skin features. Combined with multiple feature analyses, different models have their own advantages in texture patterns, maturity classification, and prediction of central sugar content.

In the domain of internal quality inspection of fruits, Huang et al. [85] combined computer vision with colorimetric sensor arrays to grade mango samples by obtaining images and odor information of mangoes. This model has a good predictive correlation for hardness and SSC. Huang et al. [86] also used computer vision to collect color information of tomatoes, obtained odor information with the help of electronic noses, and predicted the maturity and firmness of tomatoes through data fusion technology. Azadnia et al. [77] developed a visible light imaging system based on deep learning techniques, utilized data augmentation strategies to enlarge the size of dataset and conducted maturity detection and grading of hawthorn fruits. Cai et al. [87] constructed a structural light reflection imaging system to derive the direct component (DC) and alternating component (AC) images of citrus fruits. Integrated with deep learning methods, they effectively identified the early rot conditions of different citrus varieties.

To sum up, visible light imaging has the advantages of automation and efficiency [88,89], reduce labor costs [81,82], and can comprehensively analyze multiple features of fruits simultaneously [83,84,85,89,90], providing multi-dimensional data support for fruit quality inspection. However, Visible light imaging technology is vulnerable to environmental interference during the detection process. Changes in lighting conditions [87,88,91], complex backgrounds [82], fruit malformations [83] and other situations may all affect the detection accuracy. In addition, training deep learning models necessitates a substantial quantity of labeled data [90], and has high requirements for hardware devices at the same time [89], which restricts the rapid implementation and application of the models in actual production [77]. Other applications of optical inspection of fruit quality are shown in Table 1.

## 3. Electromagnetic Technology

### 3.1. Nuclear Magnetic Resonance

Nuclear magnetic resonance (NMR) relies on the spin characteristics of atomic nuclei. In a powerful magnetic field, the spinning nuclei’s energy levels divide, resulting in distinct spin states. Subsequently, when a radio-frequency pulse of a particular frequency is administered and its energy matches the energy disparity between the spin states, the atomic nucleus experiences resonant transitions [95]. During this process, the data acquired by NMR is applicable for the analysis of the structure and composition of substances [96]. Among them, chemical displacement (δ) arises as the circulation of electrons around the atomic nucleus varies the magnetic field magnitude, thereby altering the resonant frequency. The chemical displacement of atomic nuclei in different chemical environments is different, which can infer the connection mode of atoms and the chemical environment in the molecule [97]; Spin-spin coupling causes NMR spectra to exhibit fine structures, reflecting the connectivity sequence of atoms and spatial positional relationship in molecules [95,97]. After the radio frequency pulse stops, the spin system will undergo relaxation. Thereby, the molecular structure and dynamic information can be obtained by analyzing the relaxation time [95].

**Figure 4:**
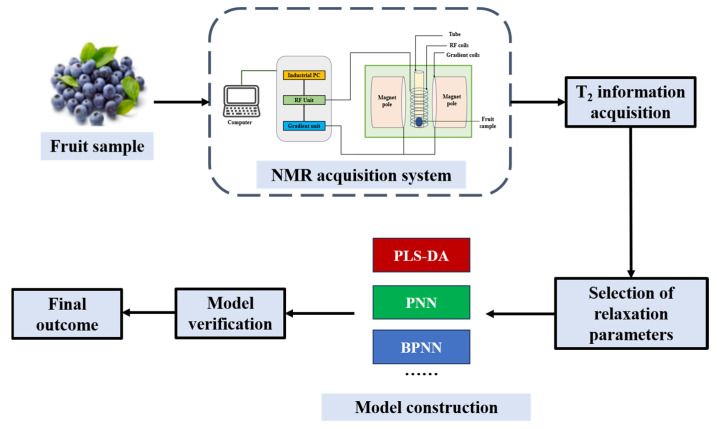
The process of detecting fruit quality using NMR technology (redesigned and redrawn from Ref. [107]).

Taking advantage of this characteristic, NMR is capable of comprehensively tracking the molecular characteristics of hundreds of metabolites in food within a single experiment, boasting excellent repeatability and precision. Among them, high-resolution one-dimensional proton NMR experiments currently dominate, and low-field nuclear magnetic resonance (LF-NMR) has potential in the analysis of oils and moisture [98,99], and is extensively applied in aspects like the identification of food components and quality evaluation, including the study of metabolomics of fruits and vegetables [100,101,102,103], analyzing the chemical composition and quality of fruit products and by-products [104,105,106]. The process of fruit quality detection by NMR technology is shown in Figure 4.

NMR can detect various quality indicators of fruits. Zhang et al. [108] measured the spin-spin NMR relaxation time and collected magnetic resonance images (MRI) of fresh pomegranates and pomegranates stored in controlled atmosphere (CA) for 3 months respectively. Through the feature analysis of MRI and the establishment of a PLS model, It is confirmed that SSC/TA has a good correlation with MRI information, and this model has certain accuracy in predicting pH and TA. Alessandra et al. [109] utilized quantitative 1H NMR and PCA analysis to study how drying influenced the polyphenols and antioxidant capacity of seven apple varieties. They found that the total phenol content was highly correlated with the antioxidant capacity, and the changes in polyphenol content were different among different varieties. The metabolite detection information obtained from this study is helpful for further understanding the nutritional value of different apple varieties. Giacomo et al. [110] analyzed the metabolites of 10 apple varieties (four kinds of carbohydrates, nine kinds of organic acids, six kinds of amino acids, rhamnitol, p-coumaroyl derivatives, phloretin/phloridzin, and choline) employing NMR spectroscopy, and PCA served to demonstrate the distinctions and similarities among varieties.

NMR technology also finds broad application in the detection of fruit damage and diseases. Qiao et al. [107] collected the Carr-Purcell-Meiboom-Gill (CPMG) sequence of blueberries with LF-NMR for acquiring T2 relaxation information. Combined with HSI technology and through the multi-threshold spectral information Segmentation (MT-SIS) algorithm, the feature selection algorithm and several models were utilized to identify the rot in blueberries. It was demonstrated that the information combination of the two could boost the detection correctness.

Giuliano et al. [111] used 1D 1H NMR spectroscopy and reverse-phase high performance liquid chromatography with diode array detection (RP-HPLC-DAD) technology to study the effects of grape red rot on the metabolites of ‘Cabernet Sauvignon’ grapes and found that the primary and secondary metabolites of grapes changed significantly after virus infection, such as modifications in the abundances of sugars, organic acids, amino acids and phenolic substances.

Wang et al. [112] studied the water distribution and migration of damaged tissue of ‘Korla’ fragrant pears by LF-NMR, and explored the light propagation characteristics by Monte Carlo simulation. It was found that the damage caused the change of water state, and the optical absorption coefficient $\mu_{a}$ and reduced scattering coefficient $\mu_{s}^{\prime }$ were related to the water state. The model founded on $\mu_{s}^{\prime }$ could effectively discriminate the damage of fragrant pears.

NMR technology can penetrate fruits to detect their internal structure and compositional information, simultaneously determine multiple metabolites to achieve multi-parameter detection [111,112,113], with high reproducibility and reliable and stable results [114]. However, for compounds with complex structures, detection and identification are difficult. For example, in the study of grapes, for complex-structured substances such as phenolic compounds, it is difficult to analyze them accurately using only 1D 1H NMR technology, and other techniques such as RP-HPLC-DAD need to be combined [111]. In addition, NMR signals are vulnerable to interference from various factors. The complex components inside fruits are prone to cause signal overlap, resulting in difficulty in analysis and affecting the accuracy of the test results [107,110,113,115]. Moreover, sample preparation and environmental factors may interfere with the signals and increase the risk of misjudgment.

### 3.2. Terahertz

**Figure 5:**
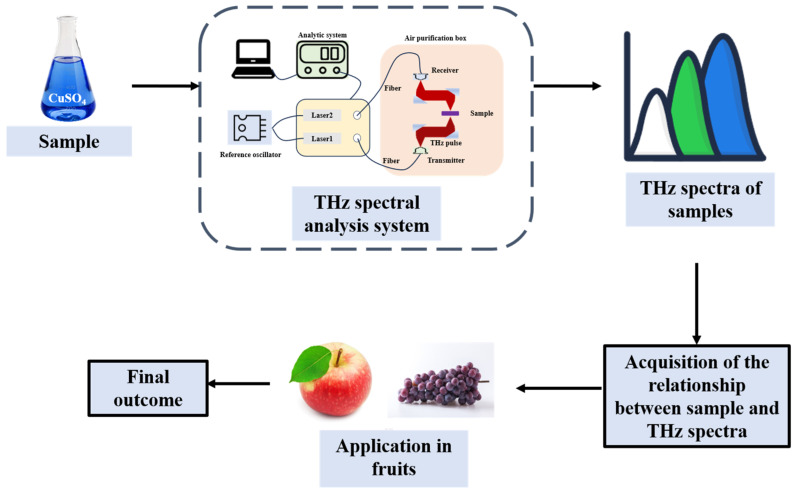
The process of detecting fruit quality using THz technology (redesigned and redrawn from Ref. [19]).

Terahertz (THz) waves refer to electromagnetic radiation within a frequency range of 0.1 to 10 THz. Owing to the high absorbency of THz waves by water molecules, along with the distinctive absorption and dispersion properties of organic molecules in this frequency range, these waves can serve the purpose of substance identification [116]. At present, THz technology is widely applied in fields such as agriculture [117,118], food quality inspection [119,120] and pharmaceutical industry [121]. To enhance the performance of THz detection, various THz metamaterials have emerged. For instance, laser-engraved self-supporting THz metamaterials are used for the rapid analysis of fruit acids [122]. Meanwhile, integrating THz detection technology with machine learning algorithms can enhance the accuracy and efficiency of detection [123,124]. The process of fruit quality detection by THz technology is shown in Figure 5.

Ren et al. [125] put forward a novel approach to monitor the variations in moisture content of pear slices using THz technology. The transmission response and path loss response of the sample were measured within the frequency range of 0.75 THz–1.1 THz through the vector network analyzer (VNA) and the Swissto12 system. The findings indicated that with the reduction of moisture content, disparities exist in the transmission and path loss responses. Subhajit et al. [126] developed sub-THz metamaterial stickers (Meta-Stickers) for non-invasive fruit ripeness detection. Meta-Stickers pasted on fruits can trigger two kinds of resonances. The maturity of fruits can be inferred by analyzing the changes in resonance frequencies. The mean normalized root mean square error (NRMSE) for the detection of persimmons, pears, and mangoes amounted to 0.54%.

THz technology can also discover pesticide remains in fruits. Copper sulfate is the main component of Bordeaux mixture and is widely used in agricultural products. Excessive use can pollute the environment and endanger human health. Tong et al. [19] developed a new type of metal grating, By combining it with THz time-domain spectroscopy technology, they used it to detect copper sulfate in fruits. This metal grating was fabricated by laser microfabrication technology and exhibits a prominent transmission peak within the 0.4–1.0 THz frequency band. Experimental results demonstrated that copper sulfate solutions of varying concentrations led to a red-shift in the transmission peak frequency. The lowest detectable concentration was 0.375 mg/L, and the relative error in the determination of apples and grapes was below 5.8%. Lee et al. [127] put forward an approach for discovering residual pesticides based on a THz time-domain spectroscopy system based on nano-metamaterials. Low-concentration methomyl molecules on the surface of fruits can be accurately detected in both transmission and reflection modes, providing a novel approach to non-destructive testing of residual pesticides.

THz waves are also widely used in detecting fruit damage. Flora et al. [128] explored the combination of millimeter waves and low THz waves for a nonlinear SVM classifier that is used to detect fruit damage. They used apples and peaches as experimental samples and found that bootstrap discriminant tree-support vector machine (BDT-SVM) successfully solved the problem that the previous binary classification SVM was unable to distinguish the healthy and damaged states of mixed fruits.

In fruit detection, the THz detection method generally has high sensitivity and is sensitive to changes in moisture and sugar content of fruits [125,126,129,130,131]. However, for extremely small amounts of molecules, the sensitivity of THz detection is insufficient. For example, it is difficult for ordinary THz time-domain spectroscopy technology to detect low-concentration copper sulfate in fruits. Metal gratings are required for recognition of such low-concentration substances [19]. The THz signal has a relatively high absorption loss when penetrating fruits, which affects the detection effect. Most of the reflected signals can only capture the characteristics of the fruit skin, and these signals make it challenging to access the internal flesh information. When detecting the maturity of fruits, it is impossible to effectively infer the fruit maturity only by relying on transmitted and reflected signals. Therefore, it is necessary to enhance the resolution of THz technology through some metamaterials, enabling it to deeply obtain the information of different layers of fruits and detect the internal damage and quality changes of fruits more accurately [126,127]. Furthermore, THz technology is vulnerable to the influence of system noise during the detection process, resulting in inaccurate extraction of spectral information [129]. THz detection also has problems such as complex equipment, high cost and high requirements for operators [19], which limits its wide application in real life.

Other applications of electromagnetic detection for fruit quality are shown in Table 2.

## 4. Acoustic Technology

### 4.1. Ultrasonic

**Figure 6:**
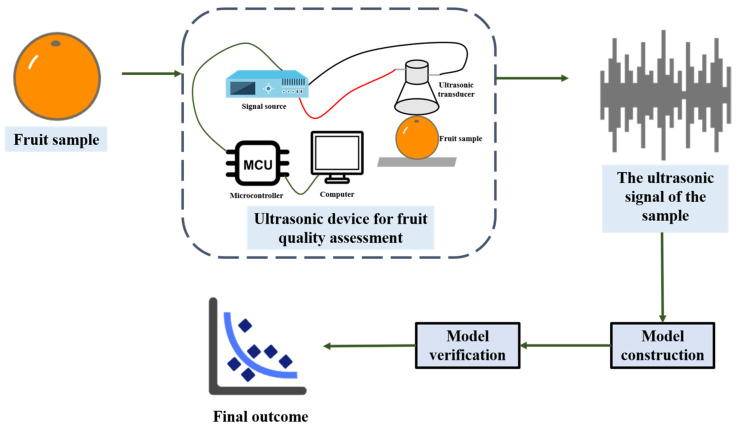
The process of detecting the quality of fruits using ultrasonic technology (redesigned and redrawn from Ref. [143]).

Ultrasonic technology uses mechanical waves with frequencies higher than the human auditory threshold (20 kHz–500 MHz) for detection [135]. The propagation properties of ultrasound within materials are intricately linked to the structural makeup of the materials [136]. Based on this principle, ultrasound technology can be used for the characteristic analysis and quality characterization of food [137]. This technology offers several benefits, including non-destructiveness, broad applicability, and low equipment cost [138]. It is extensively utilized in the food industry, for instance, in facilitating fruit drying [139,140], and removing pesticide and microbial contamination from fruits and vegetables [141,142]. For the examination of fruit quality, ultrasonic technology can be associated with various quality parameters. Among them, fruit hardness is closely related to ultrasonic characteristics, while physical and chemical indicators such as dry weight (DW), oil content, SSC, and acidity can also be measured by specific methods and combined with ultrasonic parameters. Thus, it can achieve a comprehensive assessment of fruit quality [136]. The process of fruit quality detection by ultrasonic technology is shown in Figure 6.

Numerous research results have confirmed the feasibility of ultrasonic detection of fruit hardness. Mizrach [144] measured the ultrasonic attenuation of avocados during ripening and storage and found that the attenuation changes were closely related to the changes in fruit firmness, establishing a quantitative relationship between the two. Similarly, in the studies of fruits such as mangoes [144,145] and plums [146], a significant association was also found between ultrasonic attenuation and hardness. In the research on mangoes, He [145] established a detection platform including an ultrasonic generator, transducer, filter and data acquisition system, and formulate the equation between ultrasonic attenuation and hardness. By detecting the attenuation value, the hardness of mangoes can be estimated, providing an effective basis for the assessment of mango maturity. Kim et al. [147] used specially fabricated ultrasonic transducers to test apples and observed that the apparent elastic modulus and fracture point of apples declined in a linear manner as storage duration increased. Simultaneously, the ultrasonic velocity declined, the attenuation increased, and a significant correlation existed between ultrasonic parameters and hardness. Based on this, a linear regression model with multiple variables was established to predict the hardness of apples. Morrison and Abeyratne [143] developed a single-transducer pulse-echo ultrasonic analysis technique to evaluate the overall quality of oranges by analyzing the reflected waves at the transducer-fruit boundary. The reflected energy measured by this technology is highly correlated with the hardness of oranges (R = 0.989), and this technology can non-destructively monitor the maturity of oranges and accurately predict the relative moisture content at the same time. Vasighi-Shojae et al. [148] designed a portable ultrasonic testing system for detecting the mechanical characteristics (including hardness, elastic modulus and breaking energy) of apples. Through the research on “Golden Delicious” apples, a multiple linear regression (MLR) model was formulated by combining physical and ultrasonic properties. The results show that this model exhibits a certain predictive ability in terms of the hardness, elastic modulus and rupture energy of apples.

In terms of fruit diseases, Godinez-Garcia et al. [149] put forward a non-destructive detection technique for apple larvae relying on ultrasound, wavelet analysis and statistical analysis. They took 998 Red Delicious apples as samples. By emitting 200 kHz ultrasonic waves, receiving the reflected signals and processing them with DaubechiesDB4 wavelet decomposition, they compared the statistical characteristics of the signals from healthy and infected apples and found that the signals from infected apples attenuated significantly at the center of the fruit. The ANOVA and normality tests (*p* < 0.01) confirmed the effectiveness of this method, providing a new approach for the detection of fruit pests.

However, the research of Yildiz et al. [150] pointed out that although ultrasonic testing technology has theoretical advantages, there are some problems in practical applications. When they used the ultrasonic transmission (TT) mode to test tomatoes, peaches, apples and apricots, they found that the porous and uneven surface and peel of the fruits would cause ultrasonic scattering, making it difficult for the signal to penetrate the fruits for measurement. Even when they focused the signal with an ultrasonic amplitude transformer, reliable results could not be obtained. This indicates that the current TT mode ultrasonic system has reliability issues in estimating the hardness of fruits. Moreover, Yildiz et al. proposed that in the future, the pulse-echo (PE) mode ultrasonic technique could be adopted to replace the TT mode.

Ultrasonic technology has the ability to detect multiple indicators, especially various mechanical properties [147,151], and can also reflect the internal structure of fruits [152] and thereby predicting water content and maturity [143]. Low cost is also a significant advantage of ultrasonic technology. Customized ultrasonic equipment can have a cost much lower than that of traditional ultrasonic equipment, and it is easy to operate, facilitating large-scale application in agricultural production [143]. However, the detection accuracy of ultrasonic technology is affected by multiple factors. Among them, the structure of fruits is prone to cause ultrasonic scattering, resulting in increased attenuation and making it difficult to analyze by traditional ultrasonic methods the detection results [150]. The maturity of fruits may also increase the attenuation of ultrasonic signals [153].

### 4.2. Vibration

**Figure 7:**
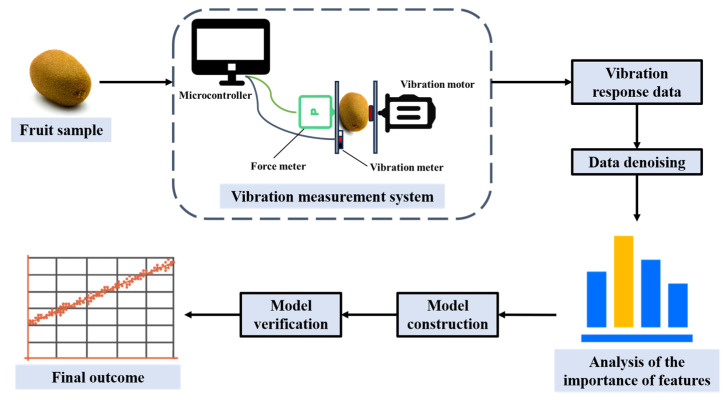
The process of detecting the quality of fruits using vibration acoustics technology (redesigned and redrawn from Ref. [156]).

Vibroacoustic detection technology achieves non-destructive and efficient fruit quality assessment by analyzing the acoustic response characteristics of fruits under controlled vibration excitation (such as frequency, amplitude and attenuation characteristics), and establishing a quantitative correlation model between these acoustic response characteristics and internal quality parameters (such as hardness, maturity and degree of tissue damage). Vibration acoustic detection methods include vibration spectrum analysis and vibration modal response method. The former detects by using the correlation between the vibration frequency of substances and their quality, while the latter analyzes the relationship between modal parameters and quality [154]. The vibration acoustic detection method is simple to operate, has low cost, and can be applied to numerous samples [155]. The process of fruit quality detection by vibroacoustic detection technology is shown in Figure 7.

In terms of the hardness detection of fruits, the relevant research achievements are remarkable. For instance, Fathizadeh et al. [157,158,159] effectively measured the hardness of apple fruits by using the acoustic vibration response method. They designed an integrated acoustic vibration fruit texture analyzer to monitor the hardness of Royal Gala apples during storage. The principal frequencies of the sound and vibration signals were analyzed through FFT, and the hardness index was calculated. Furthermore, the elastic modulus of apples was also detected in this study. The research conclusion is that acoustic signals have obvious advantages in estimating hardness, while vibration signals perform well in estimating elastic modulus. Seyedeh et al. [156] designed a non-destructive measurement device for kiwifruit hardness based on vibration analysis. After collecting the vibration response of kiwifruit, the RF model was optimized using the Bayesian algorithm, and feature selection and outlier removal were carried out. The hardness was predicted using the decision tree regression model, with an $R^{2}$ of 0.9561.

Vibroacoustic technology is also vital for the identification of fruit maturity. Zhang et al. [160] obtained the vibration response signals of kiwifruit through a vibration generator and a vibration detector, extracted the resonant frequency with FFT, and combined it with the dynamic model, successfully evaluated the changes in quality and elasticity parameter of kiwifruit during its shelf life, with $R^{2}$ ranging from 0.986 to 0.997. Chen et al. [161] developed a non-destructive acoustic detection device based on unsupervised machine learning and wavelet kernel decomposition. By tapping the surface of pineapples, analyzing the penetrating sounds, and combining advanced algorithms, it can accurately determine the maturity of pineapples. Fathizadeh et al. [159] used artificial neural network(ANN) to take the dominant acoustic frequency, dominant vibration frequency and mass of apples as features, and input them into the network in single, binary and ternary combinations respectively to estimate the shelf life of apples.

Furthermore, Zuo et al. [162] detected and graded the sugar content of watermelons by combining acoustic characteristics with machine learning methods. They designed a watermelon acoustic detection system, collected acoustic signals and conducted analysis and processing. Through PCA and stable competitive adaptive weighting algorithms, a classification model was established, and the highest accuracy rate of the verification set classification could reach 95.56%.

In the realm of internal disease detection of fruits, Liu et al. [163] established an experimental device using a micro laser Doppler vibrometer and a resonant horn. They collected the vibration response signals of jelly oranges, transformed them into vibration multi-dimensional images, and then analyze them by constructing the Resnet-Transformer network, which can accurately identify the granulation disease of jelly oranges.

In vibroacoustic detection, some self-made portable detection devices have the advantages of high speed and low cost [158,164]. Although traditional devices such as laser Doppler vibrometers have high accuracy, they also have relatively high costs and still need to be miniaturization and portability [163]. Signal processing is a major challenge in vibroacoustic detection. The accuracy, stability of the equipment, as well as factors such as environmental temperature and humidity during detection, may all cause interference to the collected signals [165,166], and noise needs to be removed through algorithms [161]. Furthermore, when establishing a prediction model based on the characteristics of different fruits, multiple factors need to be considered and the model requires optimization, which increases the difficulty of detection [156,160]. For some complex fruit quality issues, the use of vibration acoustics technology alone may not be accurate in detection. For example, when detecting apple mold heart disease, it is difficult to distinguish between mild and moderate mold heart disease samples relying only on vibration signals. Other techniques (such as Vis/NIRS technology) must be integrated to enhance the detection accuracy [167].

Other applications of acoustic detection for fruit quality are shown in Table 3.

## 5. Dielectric Property Detection Technology

**Figure 8:**
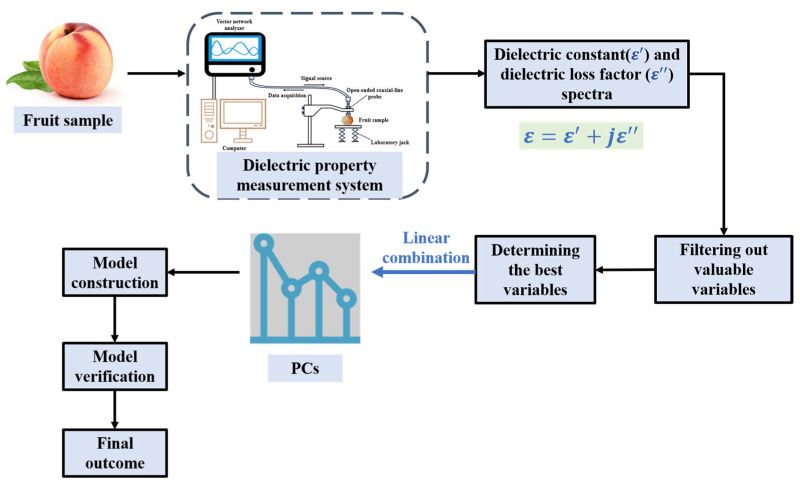
The process of detecting the quality of fruits using dielectric property technology (redesigned and redrawn from Ref. [182]).

The core principle of dielectric property detection technology lies in the mechanism of interaction between the material and the applied electric field, as well as the electrical polarization and conductivity phenomena presented by the material during this process. From a principle perspective, the parameter used to describe the interaction between a substance and an electromagnetic field is the complex dielectric constant. This constant is closely linked to the internal structure and physical-chemical properties of the material. Its components include the real part and the imaginary part: the real part, the dielectric constant, has a significant impact on the distribution state of the electric field and the phase change of electromagnetic waves in an electric field environment. The imaginary part is called the loss factor, which primarily influences the energy absorption and attenuation process and defines the level of energy consumption of the material in the electromagnetic field [173]. The dielectric properties of the detected object are influenced by various factors including frequency, moisture content, inorganic salts, and fat content [174,175]. Moisture content plays the most critical role in affecting the dielectric properties of materials [176]. Dielectric-property-based moisture content detection has been widely applied in monitoring the growth status of crops [177,178,179], and can also be utilized to assess the nutritional components and quality in agricultural products [180,181]. The process of fruit quality detection by dielectric property detection technology is shown in Figure 8.

Ardhendu et al. [183] measured the dielectric properties of plants such as apples and guavas at 16 °C and 25 °C respectively, and found that their dielectric properties varied with frequency and temperature. These data can be applied in aspects such as by using the controlled environment radio frequency dielectric heating technology to control pests of fruit crops at specific frequencies. In the study of Gu et al. [184], the dielectric constant and loss factor of bananas were closely related to the efficiency and quality during their drying processing, and the dielectric properties are also related to the color change. Through this research, the improvement methods of the banana drying processing technology can be explored. Cao et al. [185] investigated the dielectric properties of peaches with skins and after peeling in the storage period. After preprocessing the dielectric spectra and dividing the sample set, they constructed the LS-SVM to predict the internal quality. They found that the model developed from the dielectric spectra of peaches with skins had a good effect on hardness estimation. The model built upon the dielectric spectra of peeled peaches predicted hardness, SSC and moisture content well, but had a relatively low accuracy in predicting TA and electrical conductivity.

Guo et al. [186] adopted the method of dielectric spectroscopy coupled with ANN to estimate the SSC of apples. They used PCA, the partial least squares method of eliminating uninformed variables (UVE-PLS), and SPA to extract characteristic variables from the original dielectric spectra, and various models such as generalized regression neural network (GRNN), SVM, and extreme learning machine (ELM) were established respectively. The results showed that the ELM-SPA model performs best in predicting the SSC of apples. Zhu et al. [182] also used a similar method for SSC prediction in peaches. PCA-ELM demonstrated the optimal prediction performance and its comprehensive performance surpassed that of the LS-SVM and back propagation neural network (BPNN) models. However, hampered by the epidermal villi of peaches, it failed to show better prediction performance. Marcin et al. [187] measured the dielectric properties of apples using coaxial OE probe and coaxial OE-A probe with an antenna, and conducted a multi-level model fitting comparison. It was found that the dielectric spectral parameters measured with the OE-A probe had a stronger correlation with the quality parameters during shelf life of apples. Specifically, low-frequency electrical conductivity exhibited a significant negative correlation with hardness, and the apple skin had a significant influence on the dielectric response.

Liu et al. [188] measured 11 dielectric parameters of healthy apples and apples with moldy heart disease, including complex impedance and dielectric loss coefficient. They used multiple sampling techniques such as synthetic minority over-sampling technique (SMOTE) and adaptive synthesis technique (ADASYN) to process imbalanced data. Then, they combined these data with six classifiers including k-nearest neighbor (KNN) and Logistic regression (LR) to establish a non-destructive testing model for apple moldy heart disease with non-balanced dielectric data. It was demonstrated that the ST combined sampling method coupled with the GBDT classifier has the best detection effect, with an accuracy rate of 95.97%. Bian et al. [189] assessed the dielectric parameters of apples suffering drop damage from different heights across different storage days to evaluate the quality of apples with damage. The results show that with the increase of storage time, hardness, density, TA, VC and moisture content of damaged apples decreased, while the browning degree and SSC/TA increased. SSC showed a trend of rising first and then falling. The prediction effect of the main components based on dielectric parameters on the density, browning degree, SSC, TA, VC and moisture content of apples exhibited relatively good, while the prediction performance for hardness and SSC/TA was relatively poor. Liu et al. [190] combined hyperspectral imaging and dielectric techniques, and used multiple classifiers to detect Apple Core Browning. Compared with a single detection method, superior results were obtained. The SVM (RBF) classifier with CCA-based dimension reduction achieved sensitivity at 99.98%, specificity at 99.70%, and an accuracy rate of 99.70%, showing the best detection effect.

Dielectric property detection technology has prominent advantages in the realm of fruit detection. This technology is easy to operate and has a fast detection speed. With the help of specific equipment, dielectric parameters can be obtained quickly [182,186]. However, its shortcomings cannot be ignored either. The shape and surface state of the samples can interfere with the detection results, giving rise to a diminishment of data repeatability and a decline in detection accuracy [182,191], which undoubtedly increases the detection cost and operational difficulty. Moreover, dielectric property detection requires specific equipment, such as open coaxial probes [192,193], impedance analyzers [192], VNAs [193,194]. Moreover, the detection process is rather complex and has high technical requirements for operators.

Other applications of dielectric property detection for fruit quality are shown in Table 4.

## 6. Electronic Nose

**Figure 9:**
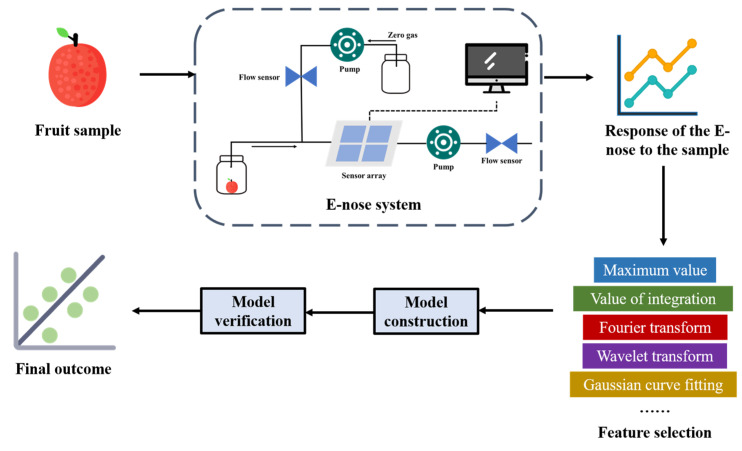
The process of detecting the quality of fruits using electronic nose technology (redesigned and redrawn from Ref. [208]).

The electronic nose (E-nose) represents a bionic olfactory analysis system. Its design inspiration comes from the human olfactory perception mechanism. It is mainly composed of a controller (core component), a gas sensor array, a signal acquisition and processing module, and a pattern recognition module. Certain E-noses may also incorporate a display unit [197,198]. Gas sensor arrays typically employ some sensitive materials, such as metal-oxide-semiconductor (MOS), conductive polymer materials (CP), electrochemical sensors (EC), quartz crystal microbalance sensors (QCM). The measurement effect is achieved through the physical or chemical reactions between the sensitive materials and the gases [198]. The signal acquisition and processing module is to collect and process data from sensors and refine it to eliminate redundancy [197]. The pattern recognition module currently has a variety of algorithms, such as KNN and SVM, used for gas recognition and classification [199]. In food industry, the E-nose is capable of detecting and identifying volatile organic compounds (VOC) in food samples [200], so it is commonly used to analyze and identify fermented food [201,202,203,204], and detect the degree of infection by pathogens in agricultural products [205,206]. It can also be used in conjunction with other detection techniques [86,207]. The process of fruit quality detection by E-nose technology is shown in Figure 9.

Mira et al. [209] used E-nose sensors to detect ‘golden delicious’ apples, reduced the data dimension by PCA, and used K-means clustering algorithm and KNN classification algorithm to classify the ripening stage of apples (divided into less mature, mature and over-mature). Qiao et al. [208] studied the discrimination between artificially ripened and naturally ripened begonia fruits. The two kinds of malus fruits are similar in appearance, but there are differences in nutritional composition. In this experiment, the self-made E-nose was employed for detection, and partial least squares regression (PLSR) was used to construct the model. It was found that the characteristic values response curves of E-nose after wavelet-transformed was highly associated with the fruit quality parameters (soluble sugar, TA, sugar-acid ratio, soluble protein, VC and SSC). It is proved that the E-nose based detection technology can effectively differentiate between artificial ripened and natural ripened begonia fruit.

E-nose has a certain application potential in aroma detection. Kouki Fujioka [210] measured apple aroma by comparing E-nose equipment via direct mass spectrometry, and conducted a comparison with sugar content and ripeness measurement values. It was found that the E-nose sensor responses increased with the cumulative intensity of apple aroma compounds measured using direct mass spectrometry, but there was no significant correlation to the sugar content and ripeness metrics. This indicates that the calyx-derived aroma measurement of an apple can be used as an indicator to identify apple characteristics. Niu et al. [211] employed the PEN3.5 E-nose to analyze the aroma of table grapes. PCA showed that the composition and concentration of aroma compounds across different varieties were significantly different, and 8 kinds of special germplasm with strong aroma were screened out. This research illustrates that the E-nose technology has the ability to effectively discriminate among different table grape varieties.

Ren et al. [212] employed an E-nose integrated with multivariate statistical analysis to classify the impact damage degree of “Fuji” apples, and used algorithms such as PCA, linear discriminant analysis (LDA), stepwise discriminant analysis (SDA), radial basis function neural network (RBFN), multi-layer perceptron neural network (MLPN) and BPNN. The results showed that MLPN and BPNN have high classification precision, indicating that the E-nose technology combined with ANN and multivariate statistics represents an effective method to classify damaged apples, but there is a problem of the signal recording time being too long. Yang et al. [213] collected E-nose data of yellow peach with different compression degrees and damage durations, used PLSR, LS-SVM and other algorithms to establish models, and found that 24 h after damage, the detection effect was good. It could determine whether the fruit was damaged, predict the extent of injury and the time when severe injury occurred.

Liu et al. [214] studied the applicability of an E-nose in detecting fungal contamination in peaches. In this experiment, three common pathogenic fungi (Botrytis cinerea, Monilinia fructicola and Rhizopus stolonifer) were used to inoculate peaches. Volatile compounds were analyzed by E-nose, combined with PCA, PLS-DA and PLSR. The results showed that the E-nose has the ability to efficiently differentiating various fungal contamination in peaches after 48 h, and the sensor response was related to terpenes and aromatic compounds. The PLSR model can effectively predict the number of fungal colonies, and the discrimination accuracy is 90%, indicating that the E-nose technology is an effective method to recognize peach fungal contamination. Xu et al. [215] employed a self-made E-nose system to identify different infection stages of citrus huanglongbing (HLB), which solved the problem of detecting zinc-deficient and HLB-positive samples, and the recognition rate reached 93.44%. The system can detect HLB at an early stage, and the recognition rate of samples at different infection stages is 93.59%.

In the domain of fruit detection, E-nose technology has unique advantages, but it also has shortcomings. Its advantage lies in the high sensitivity of the E-nose, which can acutely sense the subtle changes of volatile compounds when fruits are ripe, damaged and attacked by diseases [210,214]. Moreover, it features fast detection speed, simple operation, and can handle high-throughput testing of numerous samples rapidly [211,216,217,218,219,220,221]. It can also provide multi-dimensional information [209]. Nevertheless, the outcomes of the E-nose are easily disturbed by the environment and the fruit itself, gibing rise to the lack of consistency of the data [215,222]. For complex volatile components, accurate discrimination and quantitative analysis by E-noses may present challenges [223]. In addition, commercial equipment has limitations in detecting specific problems and in processing complex data [212,213], and this situation often requires re-modeling for different fruits.

Other applications of E-nose to detect fruit quality are shown in Table 5.

## 7. Conclusions and Prospect

From the perspective of research and application, diverse non-destructive detecting technologies have achieved rich results in fruit quality testing. Based on the analysis of fruit optical characteristics, optical technology realizes the effective detection of the intrinsic components of fruits and extrinsic characteristics. Acoustic technology provides a way to detect the mechanical properties and internal conditions of fruits through the interaction between sound waves and fruits. Electromagnetic technology uses electromagnetic radiation to obtain key information of various molecular-level substances. Dielectric properties detection technology, based on the interaction between materials and electric fields, is used for predicting the internal quality of fruits. E-nose technology, relying on the detection for VOC, has been applied in aspects such as fruit ripeness detection and disease detection.

Non-destructive testing technology has brought many significant benefits to consumers by bridging the gap between industrial quality control and the demands of end users. First of all, these technologies can precisely assess the maturity, taste and nutritional components (such as VC, sugar content) of fruits [53,210], ensuring that consumers purchase fruits with stable quality and flavor. Secondly, technologies such as terahertz spectroscopy [19] and electronic nose technology [214] can rapidly detect pesticide residues and fungal contamination, thereby enhancing the level of food safety. Thirdly, by improving the accuracy of shelf life prediction [42,208], these technologies reduce the situation of fruit spoilage after purchase, reducing waste and costs for consumers. Ultimately, integrating non-destructive testing methods into the supply chain of the fruit industry can enhance transparency and trust, enabling consumers to make informed choices when purchasing agricultural products.

However, the current implementation for fruit quality assessment using non-destructive technology still faces some challenges. Optical, acoustic and electronic nose technologies are easily interfered by environmental factors (light, noise, gas molecules). In electromagnetic technology, NMR technology has insufficient sensitivity in detection of complex compounds, and THz technology has limited penetration ability. Greatly affected by the shape and state of the sample surface, the dielectric properties lead to a decrease in detection accuracy. At the same time, there are differences in the adaptability of different technologies in practical applications. In the face of diverse fruit varieties, complex detection environments and different detection requirements, these technologies struggle to fully satisfy the demands of accurate and efficient detection. Moreover, because of the expensive equipment and complicated data processing, some advanced technologies have been limited their wide application in the fruit industry, especially among small enterprises and farmers.

In the future, the in-depth research of non-destructive evaluation in fruit quality can be carried out around the following directions:

First, multi-technology integration and collaborative innovation. Various nondestructive testing technologies have their own advantages and limitations. Through the fusion, they can complement each other and improve the detection performance. For example, near-infrared spectroscopy is suitable for surface detection, but it is not accurate enough for the location of internal small damage, while terahertz technology can detect internal defects better [130]. The combination of the two can evaluate fruit quality more comprehensively. Hyperspectral information can obtain spatial and spectral information of fruits, and electronic nose can capture volatile organic compounds produced by fruit metabolism [230]. The combination of the two can obtain physical and chemical multi-dimensional information. In practical applications, equipment integrating multiple detection technologies can be developed to obtain multiple characteristic information of fruits in parallel, and the precision and dependability of detection can be improved by comprehensive analysis through data fusion algorithms.

Second, intelligent algorithm optimization and adaptive learning. Current nondestructive testing technology faces challenges in data processing and model construction. Intelligent algorithm optimization and adaptive learning are crucial. On the one hand, more efficient feature extraction algorithms can accurately extract key features related to fruit quality from massive detection data, reduce data dimension and improve processing efficiency. For example, in hyperspectral imaging data processing, deep learning algorithms such as stacked autoencoder (SAE) are used to learn complex features in the data to avoid manual intervention and subjectivity of traditional methods [55]. On the other hand, adaptive learning can automatically adjust the parameters and algorithms according to different fruit types and detection environments to improve the generalization ability of the model. For example, DomAda-FruitDet, a domain adaptive anchor-free fruit detection model proposed by Zhang et al. [231], can detect diverse fruits like apple, tomato, dragon fruit and mango under the conditions of small targets, stacking, multi-scale and night scenes, with significantly superior performance compared to traditional anchor box models. This offers an efficient solution for non-manual labeling in smart orchards.

Third, miniaturized equipment and low-cost solutions. Many advanced nondestructive testing technologies are limited to small enterprises and farmers because of expensive equipment and complex operation. In the future, we should focus on the advancement of miniaturized, compact and cost-efficient testing equipment. For example, using MEMS technology, the detection module is integrated on a small chip to reduce the device size and cost [232]. For optical inspection devices, miniaturized spectrometers and imaging systems can be developed to make them easy to carry and inspect in the field [233]. In terms of cost reduction, the manufacturing process of the equipment is optimized, the material with low price but stable performance is selected, the operation process is simplified, and the requirements for the professional skills of the operators are reduced, so that more fruit industry practitioners can afford and facilitate the use of non-destructive testing technology, and promote its wide popularization in the industry.

Fourth, the whole industry chain digitization and standardization system construction. From orchard planting to market sales, the fruit industry involves many links, and it is strategically important to build a digital and standardized system of the whole industry chain. In the planting process, non-destructive testing technology combined with Internet of things sensors was used for real-time tracking of fruit development status to achieve precise planting management [234]. In the post-harvest processing stage, standardized non-destructive testing procedures and quality grading standards are formulated to ensure the consistency and traceability of fruit quality [235]. In the transportation and sales process, the quality information of fruits is shared in real time through the digital platform, and consumers can obtain detailed information such as the origin, picking time, and detection results of fruits by scanning two-dimensional code, which enhances consumer trust. At the same time, a unified industry standard should be established to regulate the deployment of non-destructive detection techniques and promote the standardization and sustainable development of the fruit industry.

## Figures and Tables

**Table 1:** Other applications of optical inspection of fruit quality.

Techniques	Samples	Applications	Modeling Methods	Best Accuracy/Coefficient of Determination	References
Near-infrared spectroscopy	“Millennium” cherry tomatoes	Detection of SSC and lycopene content	Mixed temperature correction model, EPO, PLS	0.8988 for SSC, 0.8023 for lycopene content	[36]
	“Fuji” apples	Detection of SSC and the degree of water core disorder	PLS	0.9808 for SSC, 0.9562 for the degree of water core disorder	[37]
	“Golden Delicious”, “Granny Smith”, “Braeburn” and “Royal Gala” apples	Detection of the distribution of internal dry matter and total sugar content	PLS	0.83 for dry matter, 0.81 for total sugar content	[39]
	Five varieties of red jujubes from Henan, Shanxi, Xinjiang, Hebei and Gansu	Variety identification	FiLDA-KNN	0.944	[40]
FT-NIRS	“Victoria” and “Autumn Royal” grapes	Investigation of the relationship between glucose, acid value and consumer preferences	PLS, PCA, Spearman correlation analysis	0.8304 for TSS	[21]
Visible/near-Infrared spectroscopy	“Fuji” apples	Detection of SSC	BP, MLR	0.87	[35]
	Persimmons	Evaluation of fruit quality by the light penetration depth in the fruit	LR	0.97 for 850 nm	[41]
	“Comte de Paris” pineapples	Detection of maturity and SSC	PCR, PLSR, ANN, KNN, PLSDA, SVMDA	0.908 for maturity, 0.7596 for SSC	[38]
	Winter jujubes	Detection of SSC of fruits at different maturity stages and prediction of the shelf life	SVR, PLSR	0.837 and 0.806 for SSC of mid-ripe and ripe winter jujube, 0.89 and 0.91 for shelf life of mid-ripe and ripe winter jujube	[42]
HSI	“Fuji” apples	Detection of hardness	PLS, SVR	0.6808	[68]
	“Touriga Franca” grapes	Detection of sugar content	PLSR, NN	0.95	[63]
	“Tempranillo” grapes	Detection of SSC and anthocyanin concentration	Epsilon-SVM	0.92 for SSC, 0.83 for anthocyanin concentration	[64]
	“Red delicious” and “Golden Delicious” apples	Detection of pH, SSC, TA and total phenol	PLS, ANN	0.9919 for pH, 0.9989 for TA, 0.9999 for SSC, 0.9989 for total phenol	[65]
	“Red Globe” grapes	Detection of SSC	PLSR based on spectral, image and fusion information	0.9762	[62]
	“Sugarone Superior Seedless”, “Thompson Seedless”, “Victoria”, “Sable Seedless”, “Alphonse Lavallée”, “Lival” and “Black Magic” grapes	Detection of SSC, total flavonoids and anthocyanins	PLS, MLR	0.97 for SSC, 0.95 for total flavonoids, 0.99 for anthocyanins	[61]
	“BiMei”, “Kula” and “Milv” melons	Detection of nitrogen and potassium in leaves, sucrose and reducing sugar in fruits	11 CNN Models	0.958 for nitrogen, 0.921 for potassium, 0.958 for sucrose, 0.936 for reducing sugar	[66]
	“Hutai 8”, “Kyoho”, “Muscat” and “Summer black” grapes	Variety identification	EEMD-DWT, CARS-SPA, SVM	0.993125	[67]
Visible light imaging	Bananas, apples	Detection of banana maturity and apple insect damage defects	ViT-shallow classifier, CNN-transfer learning	0.949 for banana maturity, 0.958 for apple insect damage defects	[92]
	16 kinds of fruits such as apples and bananas	Evaluation of fruit quality	ViT, CNN models, machine learning models, models trained for a single fruit	0.9794 for union dataset	[90]
	Apples, bananas, Oranges, papayas and guavas	Rotten fruit classification, fruit shelf life prediction	VGG16, InceptionV3 combined with CNN, Gaussian Naive Bayes, RF	0.95 for rotten fruit classification, 0.88 for shelf life prediction	[89]
	Dragon fruits	Maturity classification	ResNet18, ResNet50, ViT Tiny, ViT Small	0.91	[93]
	“Red Fuji” apples	Quality grading	Improved YOLOv5s, Swin Transformer-ResNet18	0.9446	[88]
	Apples	Quality detection	Haar Cascade classifier, CNN, machine learning models	None	[94]
	“Fengxian” apples and “Yantai” apples	Grading	Improved YOLOv5s, SSD, YOLOv4, original YOLOv5s	0.906	[91]

Abbreviations: SSC, soluble solids content; EPO, external parameter orthogonalization; PLS, partial least squares; FiLDA, fuzzy improved linear discriminant analysis; KNN, k-nearest neighbors; PCA, principal component analysis; BP, back propagation; MLR, multiple linear regression; LR, linear regression; PCR, principal component regression; PLSR, partial least squares regression; ANN, artificial neural network; PLSDA, partial least squares discriminant analysis; SVMDA, support vector machine discriminant analysis; SVR, support vector regression; NN, neural network; Epsilon-SVM, epsilon-support vector machine; CNN, convolutional neural network; EEMD-DWT, ensemble empirical mode decomposition-discrete wavelet transform; CARS-SPA, competitive adaptive reweighted sampling-successive projections algorithm; SVM, Support Vector Machine; RF, random forest.

**Table 2:** Other applications of electromagnetic detection for fruit quality.

Techniques		Samples	Applications	Modeling Methods	Best Accuracy/Coefficient of Determination	References
NMR		“Almagold” and “Golden Delicious” apples	Analysis of metabolite and network differences related to apple scab resistance	None	None	[132]
		Apples	Analysis of changes in nutritional components during the browning process	PLS-DA	None	[133]
		“Gala”, “Cripps Pink”, “Elstar”, “Boskoop”, “Braeburn” and “Holsteiner Cox” apples	Analysis of and identifying the geographical origin, variety and production method	PCA, RF	0.885 for the differentiation of German and non-German samples, 0.807 for the differentiation of regional origin within Germany, 0.795 for the differentiation of biologically and conventionally produced apples, 0.732 for the taxonomic variety	[114]
		“Red Delicious “ and “Lord Derby” apples	Internal subcellular physiological changes during the ripening and mealiness processes	None	None	[113]
High-Resolution Magic Angle Spinning NMR		“Golden Delicious”, “Rubens” and “Braeburn” apples	Metabolomic analysis and investigating the effects of different cultivation methods on apple metabolic profiles	PCA, PLS-DA	None	[115]
NMR and NIRS		Apples (cv. Elshof)	Prediction of postharvest dry matter, SSC, Hardness and Acidity	PCA, PLS	0.82 for dry matter, 0.80 for SSC	[134]
THz	Apples and pears		Detection of moisture and transmission response	None	None	[131]
Terahertz time-domain transmission spectroscopy	Apples		Detection of SSC	PLS	Higher than 0.999	[129]
NIRS, THz spectroscopy, FTIR-ATR spectroscopy, Raman spectroscopy	Mamey fruits		Study of changes in moisture ratio and carotenoid compounds during the dehydration process	Drying kinetics model, correlation between spectra and sample components	0.9998	[130]

Abbreviations: SSC, soluble solids content; PCA, principal component analysis; RF, random forest; PLS-DA, partial least squares discriminant analysis; PLS, partial least squares.

**Table 3:** Other applications of acoustic detection for fruit quality.

Techniques	Samples	Applications	Modeling Methods	Best Accuracy/Coefficient of Determination	References
Ultrasonic waves	Korean (Sansa cultivar) apples	Detection of hardness	MLR	0.990	[151]
	Korean (Sansa cultivar) apples	Analysis of softening during storage	Exponential function model, WLS	0.9701 for the high frequency signal, 0.9686 for the low frequency signal	[152]
	“YouKou” and “Fuji” apples	Prediction of hardness	MLR, PCA, ANN	0.9435 for YouKou, 0.9023 for Fuji	[168]
	“Golden Delicious” apples	Prediction of mechanical properties (hardness, elastic modulus, stiffness)	ANN	0.999 for hardness, elastic modulus and stiffness	[153]
Special non-contact ultrasonic transducer	“Fuji” apples	Detection of hardness	Multi-gaussian beam	None	[169]
Vibration spectrum	“Golden Delicious” apples	Prediction of hardness and pH value	SVR, PLSR	0.72 for hardness and 0.55 for pH value	[170]
	“Korla” pears	Detection of internal defects (brown heart)	Dominant frequency and storage time, dominant frequency and the percentage of defective mass	0.951 for dominant frequency and storage time, 0.967 for brown heart	[165]
	“Gala” apples	Prediction of hardness within the shelf life	Geometric modeling of apple fruit, finite element simulation, hardness index modeling	0.975	[171]
	“Hongyang” kiwifruits	Detection of pulp hardness, stiffness, and peel hardness	CARS-PLS	0.96 for pulp hardness, 0.95 for stiffness, 0.93 for peel hardness	[166]
Vis/NIR, vibration	“Fuji” apples	Detection of core rot	DMLPT, improved MobileNet, PLS-DA, SVM, ELM	0.9931	[167]
Laser doppler vibrometer	“Qilin” watermelons	Detection of postharvest hardness	None	None	[172]
	Pears	Detection of hardness	DA, KNN, BPNN, SMLR, PLSR	0.905 in classification, 0.832 in regression	[164]

Abbreviations: MLR, multiple linear regression; WLS, weighted least squares; PCA, principal component analysis; ANN, artificial neural network; PLS, partial least squares; CARS, competitive adaptive reweighted sampling; SVR, support vector regression; PLSR, partial least squares regression; DMLPT, dual-input multi-layer perceptron-transformer; PLS-DA, partial least squares discriminant analysis; SVM, support vector machine; ELM, extreme learning machine; DA, discriminant analysis; KNN, k-nearest neighbors; BPNN, back propagation neural network; SMLR, stepwise multiple linear regression.

**Table 4:** Other applications of dielectric property detection for fruit quality.

Techniques	Samples	Applications	Modeling Methods	Best Accuracy/Coefficient of Determination	References
Dielectric property measurement	“Early Dew”, “Honey Brew” and “Rocio” honeydew melons, watermelons, apples	Detection of sweetness and hardness	None	None	[195]
	“Fuji”, “Pink Lady” and “Red Rome” apples	Variety and internal quality Identification	LVQ, SVM, ELM	0.998	[192]
	“Red delicious” apples	prediction of apple pH and SSC	PCA-MLR, PLSR	0.7765 for pH, 1.0000 for SSC	[193]
	“Tommy Atkins” mangos	Prediction of maturity	None	None	[191]
	“Fuji” apples	Prediction of the hardness, VC, SSC, TA, and SSC/TA of fruits with static pressure damage	Regression	0.711 for hardness, 0.603 for VC, 0.608 for SSC, 0.557 for SSC/TA	[196]
Actuation by dielectric elastomer actuator, vibration	“Sun Fuji” and “Jonagold” apples	Evaluation of frequency response and hardness	Hardness index model	None	[194]

Abbreviations: SSC, soluble solids content; TA, titratable acid; LVQ, learning vector quantization; SVM, support vector machine; ELM, extreme learning machine; PCA, principal component analysis; MLR, multiple linear regression; PLSR, partial least squares regression.

**Table 5:** Other applications of E-Nose to detect fruit quality.

Techniques	Samples	Applications	Modeling Methods	Best Accuracy/Coefficient of Determination	References
E-nose	“Dabai” peaches	Detection of freshness	LR based on the maximum SNR of SR	0.85	[217]
	“Fuji” apples	Prediction of storage time	LR based on the maximum SNR of SR	0.8462	[216]
	“Fuji” apples	Prediction of low-temperature storage duration and quality (hardness, SSC, TA)	LDA, PLS, BPNN, MLPN	0.9860 for storage duration, 0.9550 for hardness, 0.9504 for SSC, 0.9860 for TA	[224]
	“Red Fuji” apples	Detection of storage period, including TA, SSC during storage	PLS	0.9063 for TA, 0.9170 for SSC	[225]
	“Royal Delicious” apples	Detection of bacterial contamination level	PCA, WHCA	0.9678 (The variance interpretation rate of PCA)	[222]
	“Fuji” apples	Detection of penicillium rot and defects	PCA, LDA, KNN, PCA-DA, PLS-DA; PLS, SI-PLS, GA-PLS, CARS-PLS	0.9722 in classification, 0.972 in regression	[226]
	“MaoYuan” apples	Detection and classification of pesticide residues	PCA, LDA, SVM	0.9732	[218]
	“Fuji” apples	Detection of fungal infections	KNN, RF, SVM, CNN, BPNN and their optimized models	0.9840	[219]
	“Red Fuji” apples	Quality grading detection system	KNN-SVM	0.9778	[220]
	“Fuji” apples	Detection of core rot	Fisher, MLPNN, RBFNN	0.8846	[221]
	“Golden Delicious” apples	Detection of early rot caused by penicillium	LDA	0.974	[227]
FT-NIRS, E-nose	“Fuji” apples	Detection of core rot	Fisher, MLPNN, RBFNN	0.877	[228]
E-nose, E-tongue	“Ralls”, “Jonagold”, “Orin”, “Indo” and “Hanfu” apples	Assessment of flavor variations and quality indicators (color, texture, SSC, TA, starch content)	LDA, PCA, HCA	0.9747 (The variance interpretation rate of LDA)	[223]
	“JinShuo” yellow peaches	Evaluation of flavor changes at different maturity stages	PCA, PLS-DA, correlation network analysis	0.782 (The variance interpretation rate of E-nose), 0.823 (The variance interpretation rate of E-tongue)	[229]

Abbreviations: SSC, soluble solids content; TA, titratable acid; LR, linear regression; SR, signal reconstruction; SNR, signal-to-noise ratio; LDA, linear discriminant analysis; PLS, partial least squares; BPNN, back propagation neural network; MLPN, multilayer perceptron; PCA, principal component analysis; WHCA, ward’s hierarchical cluster analysis; KNN, k-nearest neighbors; PLS-DA, partial least squares discriminant analysis; SI-PLS, synergy interval partial least squares; GA-PLS, genetic algorithm partial least squares; CARS-PLS, competitive adaptive reweighted sampling-partial least squares; SVM, support vector machine; RF, random forest; CNN, convolutional neural network; MLPNN, multi-layer perceptron neural network; KNN-SVM, k-nearest neighbors support vector machine; RBFNN, radial basis function neural network; HCA, hierarchical cluster analysis.

## Data Availability

The original contributions presented in this study are included in the article. Further inquiries can be directed to the corresponding authors.
